# Revisiting the mitogenomic phylogeny of Salmoninae: new insights thanks to recent sequencing advances

**DOI:** 10.7717/peerj.3828

**Published:** 2017-09-18

**Authors:** Jose L. Horreo

**Affiliations:** Department of Biodiversity and Evolutionary Biology, National Museum of Natural Sciences (CSIC), Madrid, Spain

**Keywords:** Salmonid, Salmonidae, Coregoninae, Thymallinae, Salmoninae, Evolution, Bayesian, Phylogeny, Molecular dating, RLC

## Abstract

The phylogeny of the Salmonidae family, the only living one of the Order Salmoniformes, remains still unclear because of several reasons. Such reasons include insufficient taxon sampling and/or DNA information. The use of complete mitochondrial genomes (mitogenomics) could provide some light on it, but despite the high number of mitogenomes of species belonging to this family published during last years, an integrative work containing all this information has not been done. In this work, the phylogeny of 46 Salmonidae species was inferred from their mitogenomic sequences. Results include a Bayesian molecular-dated phylogenetic tree with very high statistical support showing Coregoninae and Salmoninae as sister subfamilies, as well as several new phylogenetic relationships among species and genus of the family. All these findings contribute to improve our understanding of the Salmonidae systematics and could have consequences on related evolutionary studies, as well as highlight the importance of revisiting phylogenies with integrative studies.

## Introduction

The Salmonidae family is the only living one of the Order Salmoniformes, and it comprises three different subfamilies: Salmoninae, Coregoninae and Thymallinae. The phylogenetic relationships among them have been and continue being largely discussed: some authors claimed the existence of a sister-group relationship between Coregoninae and Thymallinae (Campbell et al., 2013; Shedko, Miroshnichenko & Nemkova, 2013; Ma et al., 2016; Macqueen & Johnston, 2014), others between Coregoninae and Salmoninae (Alexandrou et al., 2013; Near et al., 2012), and others between Thymallinae and Salmoninae (Crête-Lafrenière, Weir & Bernatchez, 2012; Yasuike et al., 2010). All these studies have been carried out during last years with different kinds of information including nuclear DNA, mitochondrial DNA, morphological characteristics, or a combination of those, but no consensus still exists regarding the Salmonidae phylogeny (e.g., Crête-Lafrenière, Weir & Bernatchez, 2012). One of the main reasons for this lack of consensus is the insufficient taxa sampling, especially in studies with increased genetic data, such as complete mitochondrial genomes (e.g., Ma et al., 2016), so the tree topology of this family would benefit from increasing sampling of taxa/species.

It is known that the use of only one or few representative genes can be useful for inferring true phylogenies (Horreo, 2012), but in this family it has not been enough probably due to processes such as genome duplication (e.g., Alexandrou et al., 2013; Macqueen & Johnston, 2014). In this sense mitogenomics, or the study of complete mitochondrial genomes, has been proposed some years ago as an useful tool for phylogenetic inferences in almost all kind of organisms, including mammals (Arnason et al., 2002), birds (Pacheco et al., 2011), insects (Wei et al., 2010), cnidarians (Kayal et al., 2013), and others. This is of course also the case of fish (e.g., Miya & Nishida, 2015), where major advances have been done in sequencing complete mitochondrial genomes last years. In the case of the Salmonidae family, several incomplete phylogenies of each of the genus belonging to it have been recently published, especially in studies describing new mitogenomes (Balakirev et al., 2016; Balakirev, Romanov & Ayala, 2017; Ho et al., 2016; Li et al., 2016; Liu et al., 2016; Ma et al., 2016; Sahoo et al., 2016; Xue & Han, 2016; Yasuike et al., 2010; Yu & Kwak, 2015). The problem of such phylogenies is the lack of species/genus on them so, in addition to the own lack of information per se for it, and as limited taxon sampling in phylogenies can provide erroneous topologies (Heath, Hedtke & Hillis, 2008), they could lead to mistakes. Therefore, the Salmonidae family do not have, to date, an integrative analysis including all the new mitogenomes published last years in order to clarify the phylogenetic relationships among all their species as well as among their genus; this was precisely the aim of this work.

## Materials and Methods

The complete mitogenome of 46 Salmonidae species (the most complete dataset to date) together with the mitogenome of two outgroups (*Umbra limi* and *Esox lucius*) were obtained from the GenBank database (accession numbers and details are shown in Table 1). From them, 12 belonged to the Coregoninae subfamily (nine, two and one mitogenomes to the Coregonus, Prosopium, and Stenodus genus, respectively), 10 to the Thymallinae subfamily (it has an only genus, Thymallus), and 24 to the Salmoninae subfamily (two, three, eight, one, seven and three to the Brachymystax, Hucho, Oncorhynchus, Parahucho, Salvelinus and Salmo genus, respectively). All these mitogenomes have been published between the years 2010 and 2017 thanks to several authors (see details in Table 1). Sequences were viewed with AliView v.1.17.1 (Larsson, 2014) and aligned with MAFFT under the algorithm G-INS-i (Yamada, Tomii & Katoh, 2016) because of its accuracy (Pais et al., 2014). The final mitogenomic alignment was 15,711 basepairs in total length.

**Table 1:** Species (and Genbank accession numbers) included in this study (family Salmonidae). Year: year published, GenBank: accession number.

Subfamily	Genus	Species	Year	GenBank
Coregoninae	Coregonus	*Coregonus peled*	2014	NC025576
Coregoninae	Coregonus	*Coregonus autumnalis*	2015	NC027277
Coregoninae	Coregonus	*Coregonus chadary*	2016	NC030175
Coregoninae	Coregonus	*Coregonus ussuriensis*	2014	NC025648
Coregoninae	Coregonus	*Coregonus muksun*	2015	NC028593
Coregoninae	Coregonus	*Coregonus lavaretus*	2016	AB034824
Coregoninae	Coregonus	*Coregonus oxyrinchus*	2012	JQ661413
Coregoninae	Coregonus	*Coregonus clupeaformis*	2013	NC020762
Coregoninae	Coregonus	*Coregonus nasus*	2013	NC020760
Coregoninae	Prosopium	*Prosopium williamsoni*	2013	NC020763
Coregoninae	Prosopium	*Prosopium cylindraceum*	2013	NC020764
Coregoninae	Stenodus	*Stenodus leucichthys*	2013	NC020761
Thymallinae	Thymallus	*Thymallus burejensis*	2015	NC027411
Thymallinae	Thymallus	*Thymallus tugarinae*	2016	KJ866483
Thymallinae	Thymallus	*Thymallus brevirostris*	2016	KJ866486
Thymallinae	Thymallus	*Thymallus mertensii*	2016	NC029216
Thymallinae	Thymallus	*Thymallus pallasii*	2015	NC027408
Thymallinae	Thymallus	*Thymallus baicalolenensis*	2016	KJ866482
Thymallinae	Thymallus	*Thymallus yaluensis*	2016	KJ866484
Thymallinae	Thymallus	*Thymallus arcticus*	2016	FJ872559
Thymallinae	Thymallus	*Thymallus thymallus*	2016	FJ853655
Thymallinae	Thymallus	*Thymallus grubii*	2017	LC168675
Salmoninae	Brachymystax	*Brachymystax tumensis*	2014	KJ730525
Salmoninae	Brachymystax	*Brachymystax lenok*	2012	NC018341
Salmoninae	Hucho	*Hucho taimen*	2014	KJ711550
Salmoninae	Hucho	*Hucho bleekeri*	2016	HM804473
Salmoninae	Hucho	*Hucho hucho*	2014	NC025589
Salmoninae	Oncorhynchus	*Oncorhynchus clarkii*	2008	NC006897
Salmoninae	Oncorhynchus	*Oncorhynchus nerka*	2016	EF055889
Salmoninae	Oncorhynchus	*Oncorhynchus tshawytscha*	2016	AF392054
Salmoninae	Oncorhynchus	*Oncorhynchus masou*	2016	KU523579
Salmoninae	Oncorhynchus	*Oncorhynchus mykiss*	2015	LC050735
Salmoninae	Oncorhynchus	*Oncorhynchus keta*	2016	AP010773
Salmoninae	Oncorhynchus	*Oncorhynchus kisutch*	2016	EF126369
Salmoninae	Oncorhynchus	*Oncorhynchus gorbuscha*	2016	EF455489
Salmoninae	Parahucho	*Parahucho perryi*	2014	NC021651
Salmoninae	Salvelinus	*Salvelinus fontinalis*	2010	NC000860
Salmoninae	Salvelinus	*Salvelinus alpinus*	2010	NC000861
Salmoninae	Salvelinus	*Salvelinus leucomaenis*	2014	KF974452
Salmoninae	Salvelinus	*Salvelinus malma*	2014	KJ746618
Salmoninae	Salvelinus	*Salvelinus curilus*	2014	NC024585
Salmoninae	Salvelinus	*Salvelinus albus*	2015	NC028018
Salmoninae	Salvelinus	*Salvelinus kuznetzovi*	2016	NC029877
Salmoninae	Salmo	*Salmo salar*	2015	LC012541
Salmoninae	Salmo	*Salmo trutta fario*	2015	KT633607
Salmoninae	Salmo	*Salmo trutta trutta*	2016	AM910409
–	Umbra	*Umbra limi*	2015	KP013095
–	Esox	*Esox lucius*	2012	AP004103

In Salmonidae, complex evolutionary processes such as genome duplication have occurred, and it have influenced on its phylogenetic reconstructions (Alexandrou et al., 2013), probably because the existence of different evolutionary rates of branches (Leong et al., 2010). In cases like this, a random local clock (RLC) model, which has rate heterogeneity into account, must be employed for inferring past evolutionary histories, if not phylogenetic reconstructions can be erroneous (King & Lee, 2015). For this reason, the Salmonidae phylogeny was reconstructed under a Bayesian inference with the BEAST v.2.4.6 software (Bouckaert et al., 2014) employing the mentioned RLC, a GTR model for sequence evolution (previously estimated with jModelTest; Posada, 2008), a Birth-Death model prior, and 15 million of MCMC chain length for ensuring their convergence. Molecular clock analyses/dating were done following fossil evidences that placed the Salmonidae origin 51.80 million years (Mya) ago (Greenwood et al., 2005; Ma et al., 2016; Near et al., 2012). Tracer v 1.6 (http://tree.bio.ed.ac.uk/software/tracer/) was employed for ensuring the convergence of MCMC chains (searching for ESS > 200), and FigTree v.1.4.2 (http://tree.bio.ed.ac.uk/software/figtree/) for drawing the phylogenetic tree.

## Results and Discussion

**Figure 1:**
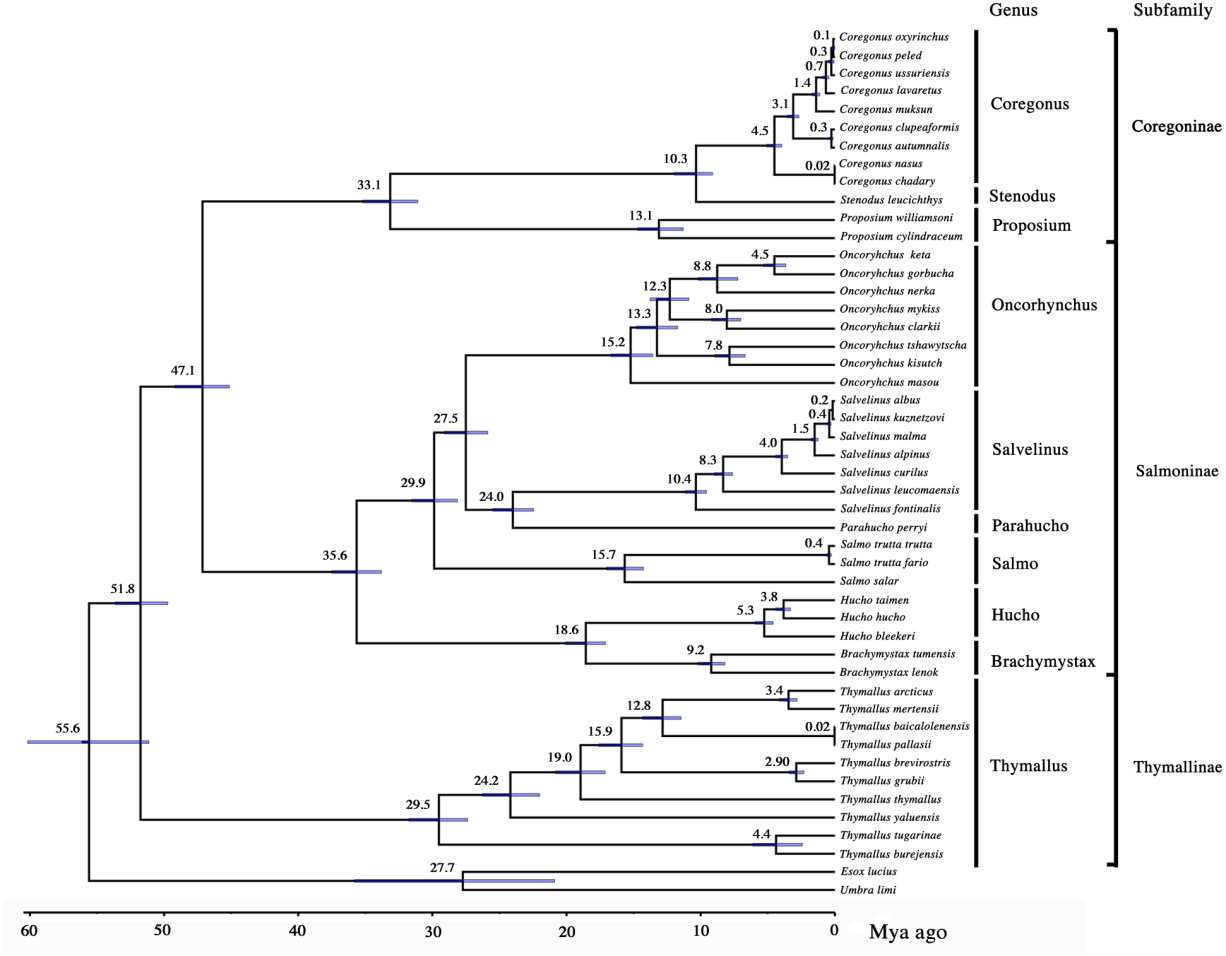
Phylogenetic relationships among subfamilies of the Salmonidae family. Node numbers show node ages, and node bars show 95% CI. All posterior probabilities (pp) = 1 in all nodes but in the *Coregonus peled*/*C. oxyrinchus* common ancestor node (pp = 0.74). Mya ago, million years before present.

The phylogenetic tree of Salmonidae (Fig. 1) showed very high statistical support with posterior probabilities (pp) = 1 in all nodes but in the *Coregonus peled*/*C. oxyrinchus* common ancestor node (pp = 0.74). The here presented topology resolved with mitogenomic sequences Coregoninae and Salmoninae as sister subfamilies with a common ancestor 47.1 (95% CI [45.1–49.2]) Mya ago, as some authors have previously proposed with nuclear genes (Near et al., 2012), as well as combining DNA and morphological data (Alexandrou et al., 2013). Other studies with mitogenomic information (Campbell et al., 2013; Ma et al., 2016) or nuclear genes (Macqueen & Johnston, 2014) had proposed Thymallinae and Coregoninae as sister subfamilies, and others employing mitochondrial (Shedko, Miroshnichenko & Nemkova, 2013; Yasuike et al., 2010) and mito-nuclear DNA (Crête-Lafrenière, Weir & Bernatchez, 2012) had proposed Thymallinae and Salmoninae as the sister ones. This study, the only done with the mitogenome of 46 different salmonid species, therefore supports Coregoninae and Salmoninae as the sister groups within the Salmonidae family, but also provides new insights into the phylogenetic relationships among genus and species of this family, including node molecular molecular node dating (Table 2).

**Table 2:** Molecular node dating (in million years ago; Mya) of the different genus belonging to the Salmonidae family estimated with Bayesian RLC inference.

Genus	Age (Mya)	95% CI
Coregonus	4.5	3.9–5.0
Stedonus	10.3	9.1–11.9
Proposium	33.1	31.8–35.2
Oncorhynchus	15.2	13.5–16.6
Salvelinus	10.4	9.5–11.1
Parahucho	24.0	22.4–25.5
Salmo	15.7	14.2–17.0
Hucho	5.3	4.6–5.9
Brachymystax	9.2	8.1–10.1
Thymallus	29.5	27.3–31.7

### Coregonidae

Within the Coregonidae subfamily (33.1 Mya ago; 95% CI [31.0–35.2]) the genus Prosopium showed an evolutionary line that diverged earlier from the Stenodus/Coregonus line. Within this last genus (Coregonus), more species than the previously published with mitogenomic information were included in these analyses (Li et al., 2016; Sahoo et al., 2016; Xue & Han, 2016), so new phylogenetic relationships among species were unravelled with mitogenomic information. *Coregonidae nasus* and *C. chadary* shared a common ancestor in one evolutionary line that diverged earlier from the other species, similarly to Xue & Han (2016)*.* After them, *C. clupeaformes* and *C. autumnalis* showed also a common and divergent evolutionary line, as Li et al. (2016) and Sahoo et al. (2016) proposed.

### Salmoninae

The sister Subfamily to Coregonidae was, as mentioned above, Salmoninae (35.6 Mya ago; 95% CI [33.7–37.5]). The topology of their different genus (Brachymystax, Hucho, Parahucho, Oncorhynchus, Salmo and Salvelinus; no mitogenomes still exists for Salvethymus) and species differed from the previously published with mitogenomic information (Ma et al., 2016; Sahoo et al., 2016). Despite this, some similarities were found with such studies, as the fact that Hucho and Brachymystax are sister genus sharing a common evolutionary line that diverged earlier than the others of the subfamily. In contrast to such studies, Parahucho was the sister genus of Salvelinus, sharing both of them an evolutionary line with a common ancestor with Oncorhynchus, being the genus Salmo placed between these last three genus (Oncorhynchus, Salvelinus, Parahucho) and the others (Hucho, Brachymystax). Within each of the Salmoninae genus, the phylogenetic relationships among species were somewhat different to those published (Balakirev et al., 2016; Campbell et al., 2013; Ma et al., 2016; Sahoo et al., 2016). For example, within the Salvelinus genus, the phylogenetic relationship of *Salvelinus fontinalis* and *S. leucomaensis* was similar to the Ma et al. (2016) one, both of them belonging to different evolutionary lines, but different from the Sahoo et al. (2016) and Balakirev et al. (2016), where they shared a common evolutionary line. The other species of the genus showed similar phylogenetic relationships to those of Balakirev et al. (2016). Within the Onchorrynchus genus, phylogenetic relationships among species were similar to those of Sahoo et al. (2016), but different to others (e.g., Campbell et al., 2013; Ho et al., 2016; Li et al., 2016; Ma et al., 2016; Yu & Kwak, 2015). In the case of the genus Hucho, Brachymystax, Parahucho, and Salmo, the phylogenetic relationships among species were similar to those inferred by Balakirev et al. (2016).

### Tymallinae

Within the Tymallinae Subfamily (29.5 Mya ago; 95% CI [27.7–31.7]), the tree showed different topology to all the other studies (Balakirev, Romanov & Ayala, 2017; Li et al., 2016; Liu et al., 2016; Ma et al., 2016; Sahoo et al., 2016). On it, the earliest diverged lineage included the *T. burejensis*/*T. tugarinae* clade, followed by the lineage of *T. yaluensis*, another including *T. thymallus*, and a last one that grouped all the other species of the genus. In contrast to all these differences in topology, *T. arcticus* and *T. mertensii* were sister species as recently proposed by Liu et al. (2016) and Balakirev, Romanov & Ayala (2017).

## Conclusions

This work integrates new mitogenomic data into the Salmoninae phylogeny, showing new phylogenetic relationships among different species and genus, but also unravelling the relative position of its subfamilies with high statistical support (almost all nodes with pp = 1) under Bayesian RLC analyses. Coregoninae and Salmoninae are, according to these results, sister subfamilies into an evolutionary line that shares a common ancestor with Thymallinae 51.8 (95% CI [49.7–53.6]) Mya ago. To date, only subsets of species or genus within the family have been published, focused on the inclusion of new mitogenomics data into the phylogenies of their genus, but no integration of all of them had been done. The new phylogenetic information here presented contributes to an advance in the knowledge of the Salmonidae systematics and in the evolutionary studies based on it, as for example the study of complex migratory behaviour (e.g., Alexandrou et al., 2013; Macqueen & Johnston, 2014). This work also highlights the importance of integrative studies in order to update the phylogenetic knowledge of families, genus, etc., with new and more complete DNA information and taxon sampling, which can unravel new phylogenetic and evolutionary relationships within them.

##  Supplemental Information

10.7717/peerj.3828/supp-1 Supplemental Information 1 Salmonidae alignment Salmonidae mitogenomic alignment done with MAFFT under the G-INS-i algorithm.

## Additional Information and Declarations

### Data Availability

The following information was supplied regarding data availability: The raw data has been provided as Supplemental File.
